# Factors Associated with Loss-to-Follow-Up during Behavioral Interventions and HIV Testing Cohort among Men Who Have Sex with Men in Nanjing, China

**DOI:** 10.1371/journal.pone.0115691

**Published:** 2015-01-05

**Authors:** Weiming Tang, Xiping Huan, Ye Zhang, Tanmay Mahapatra, Jianjun Li, Xiaoyan Liu, Sanchita Mahapatra, Hongjing Yan, Gengfeng Fu, Jinkou Zhao, Chenghua Gu, Roger Detels

**Affiliations:** 1 Guangdong Provincial Center for Skin Disease and STI Control, Guangzhou, Guangdong, 510095, China; 2 Jiangsu Provincial Central for Disease Control and Prevention, Nanjing, Jiangsu, 210009, China; 3 University of North Carolina Project-China, Number 2 Lujing Road, Guangzhou, 510095, China; 4 Department of Epidemiology, School National Institute of Cholera and Enteric Diseases, Kolkata, West Bengal, India; 5 Strategy, Investment and Impact Division, The Global Fund to Fight AIDS, Tuberculosis and Malaria, Geneva, Switzerland; China Medical University, China

## Abstract

**Background:**

Behavioral interventions (BIs) remained the cornerstone of HIV prevention in resource-limited settings. One of the major concerns for such efforts is the loss-to-follow-up (LTFU) that threatens almost every HIV control program involving high-risk population groups.

**Methods:**

To evaluate the factors associated with LTFU during BIs and HIV testing among men who have sex with men (MSM), 410 HIV sero-negatives MSM were recruited using respondent driven sampling (RDS) in Nanjing, China during 2008, they were further followed for 18 months. At baseline and each follow-up visits, each participant was counseled about various HIV risk-reductions BIs at a designated sexually transmitted infection (STI) clinic.

**Results:**

Among 410 participants recruited at baseline, altogether 221 (53.9%) were LTFU at the 18-month follow-up visit. Overall, 46 participants were found to be positive for syphilis infection at baseline while 13 participants were HIV sero-converted during the follow-up period. Increasing age was less (Adjusted Odds Ratio(aOR) of 0.90, 95% confidence Interval (CI) 0.86–0.94) and official residency of provinces other than Nanjing (AOR of 2.49, 95%CI 1.32–4.71), lower level of education (AOR of 2.01, 95%CI 1.10–3.66) and small social network size (AOR of 1.75, 95%CI 1.09–2.80) were more likely to be associated with higher odds of LTFU.

**Conclusion:**

To improve retention in the programs for HIV control, counseling and testing among MSM in Nanjing, focused intensified intervention targeting those who were more likely to be LTFU, especially the young, less educated, unofficial residents of Nanjing who had smaller social network size, might be helpful.

## Introduction

Given that HIV is still incurable, non-availability of a safe and effective vaccine, limited access to anti-retroviral treatment (ART) and declining international fund, till date, preventive approaches through behavioral interventions (BIs) and voluntary counseling and testing (VCT) remain the cornerstone of HIV control [1]–[4]. Researchers have argued that a considerable reduction in HIV transmission could be successfully achieved through a combined effect of wide spread and sustained behavioral changes by appropriate counseling of a good number of potentially high risk individuals [4], [5]. Analysis of data from three countries around the world having different cultures and diverse HIV epidemics: Uganda, Senegal and Thailand, clearly indicated that behavioral changes were successful in some containment of HIV epidemic [6]–[10]. Likewise, VCT has been also effective in motivating people to change their risky behaviors and previous meta-analyses demonstrated that VCT recipients were less likely to engage in unsafe sex than VCT non-recipients [11], [12]. While the UNAIDS report 2012 [13] revealed an increasing trend towards uptake of HIV testing globally, it was estimated that only half of all people living with HIV (PLHIV) knew their HIV sero-status and probably HIV prevention programs were not adequately reaching the population groups at highest risk. Globally, population at highest risk like female sex workers (FSW), men who have sex with men (MSM) and injecting drug users (IDUs) continue to be disproportionately affected by HIV [1], [13], [14]. It was estimated that odds of having HIV infection among MSM on average was 13 times more compared to general population in any capital city of the world [13]. Although prior research indicated that counseling and BIs were effective in reducing high risk sexual behavior among MSM [15]–[17], worldwide the median coverage of risk reduction programs among MSM was estimated to be 55%, only few countries reported 75% consistent condom use and knowledge about their own HIV sero-status was reported to be exceptionally low among MSM [13].

China has also observed a similar pattern in HIV epidemic among MSM population in recent years. The estimated HIV prevalence among Chinese MSM was found to be quite high [14], [18]–[21]. At the end of 2011, the estimated number of PLHIV was 780,000, 48,000 got newly infected and 17.4% of PLHIV were infected through homosexual route in China [18], [20]. Approximately 18 million men were estimated to be involved in homosexual activities in 2011 and HIV transmission between homosexuals rose from 0.3% before 2005 to more than 13.7% in 2011 [22]. Even though the Chinese government recently recognized the emerging burden of HIV in MSM communities and emphasized the need to expand the coverage of BIs and HIV testing among them, surveillance and targeted prevention strategies among these men were still under-developed and non-specific [23]. An analysis of 2011 surveillance data revealed that approximately 77% MSM were covered by prevention programs, 74% reported using a condom during the last sexual act and only half of them were tested for HIV and were aware of their sero-status [18]. Major public health challenges of the emerging HIV epidemic among MSM were delayed national response to the HIV epidemic among MSM until 2007, lack of experienced public health workers being engaged in delivering effective intervention to this hard-to-reach population, unexplored bisexual behaviors as many had traditional marriage to hide their homosexuality while continuing to have a secret same sex relationship, poor societal acceptance of same sex behavior leading to rapid congregation of MSM communities in different venues of metropolitan areas like sex clubs, bars, saunas, bathhouses, public toilets and parks which promoted homosexual prostitution, erotic activities and practice of unprotected sex and lastly under utilization of available HIV prevention services [19], [23], [24]. Furthermore, most of the previous studies involving MSM in China had focused on assessing factors associated with HIV risk and evidence-based intervention studies targeting this group were less documented [24], [25]. To the best of our knowledge, till date only three published papers have documented the role of preventive intervention strategies among MSM in China. Out of these three BIs, two peer-based communication programs regarding safer sex behaviors were effective in promoting condom use and HIV testing among MSM in Chengdu [26] and Anhui province of China [27] while the efficacy of another internet-based HIV.

BIs among MSM in Hong Kong was inconclusive [28]. Although BIs and counseling were effective in altering high risk sexual behaviors, acceptance of these prevention services among MSM was low. To improve the acceptance of such programs among MSM, comprehensive understanding of the predictors of being loss-to-follow-up (LTFU) among MSM seemed crucial.

The objective of this study was to identify socio-demographic and behavioral characteristics of MSM who were LTFU during BIs and HIV testing among HIV sero-negative MSM attending a designated sexually transmitted infection (STI) clinic of the Jiangsu Provincial Center for Disease Control in Nanjing, China, so that adequate counseling targeting these at-risk groups could help in the improvement of retention in HIV control programs.

## Methods

### Study Design and Sampling Methods

Details of the sampling methods and recruitment of the participants were described elsewhere [29], [30]. Briefly, respondent-driven sampling (RDS) was used to recruit the eligible and consenting participants. Male persons, aged 18 years or more and reported to have had oral and/or anal sex with men during the past 12 months, who were HIV sero-negative, did not participate in a similar survey within the past 3 months, willing to complete the 18-month follow-up study and provided informed consent were eligible for the study. Overall, 10 seeds having diverse characteristics in terms of income, age, occupation, and “cruising areas” were recruited. Interviews were conducted and blood samples were collected at the STI clinic of the Jiangsu Provincial Center for Disease Control in Nanjing.

### Follow-up visits and intervention

Written informed consent was obtained from all eligible participants before the interview and blood collection in each visit. A face-to-face interview was conducted using an interviewer administered structured questionnaire by trained professionals in a separate and private room. At the end of the interview, specific risk reduction counseling was provided to each participant on HIV and other STIs (like increase in condom use/reducing number of sexual partner/avoiding high risk sexual practices like UVI/UAI/risk of alcohol/drug use during sex etc.) by an experienced counselor. Blood sample was collected next from each consenting participant for HIV and syphilis testing. Post-test counseling was provided to each subject when they returned to the clinic to collect HIV test results. Participants were asked to come back to the designated clinic for the follow-up assessment every 6 months. Any participant who was found to be HIV sero-positive at baseline or during any follow-up visits were excluded from the cohort and were linked to national free anti-retroviral treatment center.

### Retention plan

After the participants were enrolled, the phone number, email address and QQ number of them were collected. In addition, appointments regarding the next visit/follow up were scheduled, while reminding cards which includes the date of the next appointment were given them. Before two weeks of the scheduled follow up time, a reminding message was send to every eligible HIV-negative participant through texted message, email and QQ. If we did not get any responses from the participant one week before the appointed time, an additional call was made to him.

If the participant missed the scheduled appointment, two more reminding messages were delivered to him. Also, two calls were made to him one/two weeks after the appointment. If needed, new appointments were. We continued to call them 1–2 months after the initial interview, to ask whether they are willing to attend the follow up. If they are not willing to attend the follow up scurvies again, the detailed reasons for the recursion were asked and recorded.

### Measures

Information was collected on socio-demographic factors (age/marital status/official residency/education/income/whether student or not), behavioral information (sexual orientation/history of unprotected vaginal/anal intercourse (UVI/UAI) in the past six months). In our study, the size of the social network was determined on the basis of the reported number of MSM known to the participants (familiar with face/name/nickname/had his contact information and could get in touch with him within the next month). Information was also collected on self-reported history (yes/no) on any of the following symptoms: burning sensation during urination/genital discharge/genital ulcer and if a participant reported any one of the aforementioned symptoms, then he was considered to be suffering from one or more STIs.

The participants were categorized into two groups: 1) consistent participation: participants who attended the baseline survey and had returned for the follow-up visits during 6th, 12th and 18th months to the designated STI clinic; 2) non-consistent participation/LTFU: participants who attended the baseline survey but did not return for one or more follow up visits to the designated STI clinic during the 18-month follow up period. The participants who were tested syphilis positive but remained HIV negative were retained in the study.

Cruising areas/venues, HIV knowledge, and coverage of HIV preventive services have been reported previously [29], [30]. Briefly, cruising areas/venues were categorized as conventional venues (gay-bars/parks/massage-parlors/spas/saunas/internet) and non-conventional venues (specifically, meeting partners on campuses/introduced by friends, etc.).

### HIV and syphilis testing

Five ml of venous blood sample was collected from each participant for HIV and syphilis testing. Initially samples were screened for HIV using a rapid test (Acon Biotech Co. Ltd). Samples positive at screening were confirmed by Western blot (HIVBLOT 2.2, Genelabs Diagnostics, Singapore). Samples positive for Western blot were considered as HIV sero-positive. Samples were also screened for syphilis using the Rapid Plasma Reagin test (RPR: Beijing Wantai Biological Pharmacy Enterprise Co. Ltd) and confirmed by the Treponema Pallidum Particle Agglutination assay (TPPA: Livzon Group Reagent Factory). Syphilis positivity was defined as “current” when both TPPA and RPR were positive. Participants were asked to return to the designated lab to collect the test results.

### Ethical approval

The study protocol was approved by the Institutional Review Board of the Jiangsu Provincial Center for Disease Control and Prevention (JSCDC). A signed informed consent was obtained from each participant prior to the interview and blood collection. Each participant had the discretion to freely decline or withdraw from this study at any given point of time.

### Data analysis

Data was double-entered using EpiData 3.0 [31] and multiple logic checks were used to ensure the data quality. SAS version 9.1 [32] was used for all statistical analyses. Descriptive analyses were conducted to determine the distribution of socio-demographic characteristics, sexual behaviors, coverage of HIV/STI related prevention services and compared between two groups of participant (those who were LTFU & those who were retained in the study). In addition, to assess the strength and direction of associations between LTFU and potential correlates, both bivariate and multivariate regression analyses were performed [expressed in Odds Ratio (OR) & 95% confidence interval (CI)]. The main outcome variable was LTFU to BIs and predictor variables were age, marital status, official residency, education, income, occupation, sexual orientation, UAI, UVI, HIV-related knowledge, coverage of HIV/STI preventive services, STI-related symptoms, cruising venues, social network size, and syphilis positivity. Variables with a *p*-value less than 0.3 in bivariate analyses were included in the multivariate analyses.

## Results

### Demographics, behaviors, STI related symptoms and syphilis positivity

**Figure 1:**
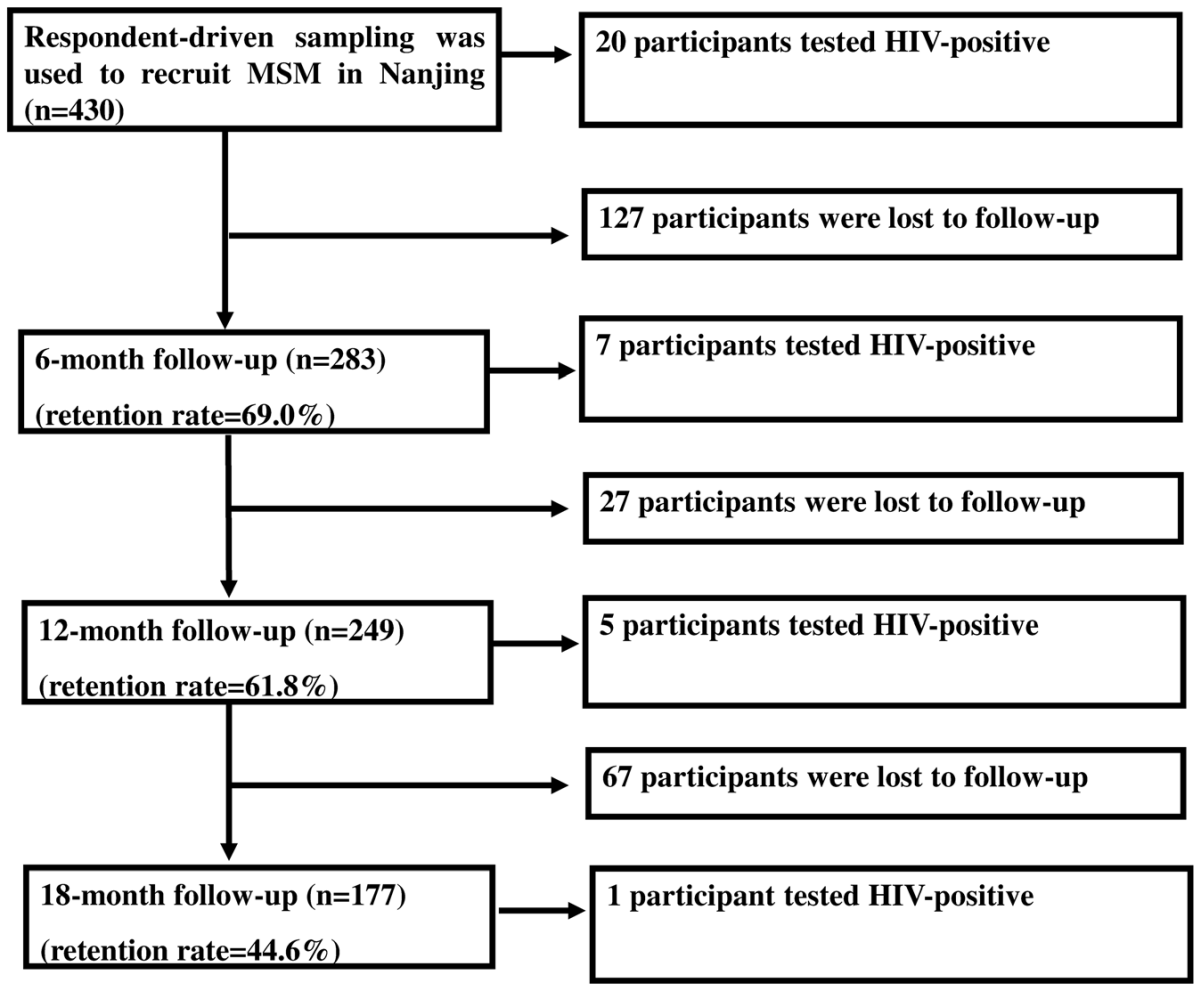
Flowchart of the Follow Up among MSM in Nanjing, China, 2008–2010.

Overall, 430 eligible MSM of Nanjing were screened at the baseline. Of these subjects, 20 were HIV sero-positives at the baseline and were excluded from the study, 410 HIV sero-negatives were invited to come to the designated clinic for the follow-up visits at 6-month, 12-month and 18-month. Of the 410 participants, 189 (46.1%) came for all the three and 221 (53.9%) missed one or more follow-up visits. 13 (6.9%) participants were sero-converted for HIV infection during the follow-up period (Fig. 1).

Overall, the mean age was 28.3±8.6 years, 61.2% of the participants were of age 20–29 years, 76.1% were never married, 59.5% were official residents of Nanjing, 68.3% attended college or above, 28.8% had a monthly income less than 1,000 Chinese Yuan (1 US Dollar = 6.12 Chinese Yuan), 29.5% were students, 57.6% contacted their casual partner through the internet and 87.1% received HIV/STI related prevention services during the past 1 year, 60.0% and 16.8% of them had UAI and UVI, respectively, during the past 6 months preceding the baseline survey. In addition, about 36.8% of the eligible participants reported that they have social network size less than 10. Overall, 24.6% reported one or more STI related symptoms and 11.2% were tested positive for syphilis infection. Socio-behavioral characteristics of the participants who attended all the follow-up visits were significantly different from those who were LTFU, as evident from the non-overlapping 95% CIs (Table 1).

**Table 1:** Demographic characteristics, behaviors, STI related symptoms and proportion of syphilis infection among HIV sero-negative MSM at baseline in Nanjing, China, 2008(N = 410).

Characteristic		Loss-to-follow-up (n = 221)			Consistent participation (n = 189)			Overall	
		N	%	95% CI	N	%	95% CI	N	%
Age (years)	*Less than 20*	23	10.4	6.4–14.5	1	0.5	0.0–1.6	24	5.8
	*20–29*	146	66.1	59.8–72.4	105	55.6	48.4–62.7	251	61.2
	*30–39*	38	17.2	12.2–22.2	45	23.8	17.7–29.9	83	20.2
	*40–49*	13	5.9	2.8–9.0	29	15.3	10.2–20.5	42	10.2
	*50 or above*	1	0.4	0.0–1.3	9	4.8	1.7–7.8	10	2.4
Marital status	*Single*	179	81.0	75.8–86.2	133	70.4	63.8–76.9	312	76.1
	*Married*	38	17.2	12.2–22.2	41	21.7	15.8–27.6	79	19.3
	*Divorced or Widowed*	4	1.81	0.0–3.6	15	7.9	4.0–11.8	19	4.6
Official resident (Hukou)	*Nanjing*	107	48.4	41.8–55.1	137	72.5	66.1–78.9	244	59.5
	*Other cities in Jiangsu*	52	23.5	17.9–29.2	32	16.9	11.5–22.3	84	20.5
	*Other provinces*	62	28.0	22.1–34.0	20	10.58	6.2–15.0	82	20.0
Education	*High school or below*	81	36.6	30.2–43.0	49	25.9	19.6–32.2	130	31.7
	*College or above*	140	63.4	57.0–69.8	140	74.1	67.8–80.4	280	68.3
Income	*Less than 1000*	59	30.9	24.3–37.5	43	26.4	19.5–33.2	102	28.8
	*1000–4000*	109	57.1	50.0–64.2	97	59.5	51.9–67.1	206	58.2
	*4000 or above*	23	12.0	7.4–16.7	23	14.1	8.7–19.5	46	13.0
Cruising venues	*Internet*	121	54.8	48.1–61.4	115	60.8	53.8–67.9	236	57.6
	*Pub, Disco, Tearoom, or Club*	46	20.8	15.4–26.2	17	9.0	4.9–13.1	63	15.4
	*Spa or bathhouse, Sauna*	29	13.1	8.6–17.6	32	16.9	11.5–22.3	61	14.9
	*Park, Public Restroom, or Public Lawn*	8	3.6	1.1–6.1	11	5.8	2.4–9.2	19	4.6
	*Other*	17	7.7	4.2–11.2	14	7.4	3.6–11.2	31	7.6
Student	*Yes*	66	29.9	23.79–36.0	55	29.1	22.6–35.6	121	29.5
	*No*	155	70.1	64.0–76.2	134	70.9	64.4–77.4	289	70.5
Coverage of HVI/STI prevention services	*Yes*	189	85.5	80.8–90.2	168	88.9	84.4–93.4	357	87.1
	*No*	32	14.5	9.8–19.2	21	11.1	6.6–15.6	53	12.9
Unprotected anal sex	*Yes*	130	58.8	52.3–65.4	116	61.4	54.4–68.4	246	60.0
	*No*	91	41.2	34.6–47.7	73	38.6	31.6–45.3	164	40.0
Unprotected vaginal sex	*Yes*	39	17.6	12.6–22.7	30	15.9	10.6–21.1	69	16.8
	*No*	182	82.4	77.3–87.4	159	84.1	78.9–89.4	341	83.2
Reporting any STI related symptoms	*Yes*	53	24.0	18.3–29.7	48	25.4	19.1–31.7	101	24.6
	*No*	168	76.0	70.3–81.7	141	74.6	68.3–80.9	309	75.4
Syphilis	*Yes*	23	10.4	6.4–14.5	23	12.2	7.5–16.9	46	11.2
	*No*	198	89.6	85.5–93.6	166	87.8	83.1–92.5	364	88.8

### Correlates of LTFU

Both crude and adjusted models revealed that age of the participant was a significant predictor of LTFU (crude Odds Ratio (COR) of 0.93, 95% CI 0.90–0.95, adjusted Odds Ratio(AOR) of 0.90, 95% CI 0.86–0.94). In the unadjusted model and after controlling all other variables, LTFU was more frequent among MSM who were official residents of the other provinces than participants from Nanjing (COR of 3.97, 95% CI 2.26–6.98, AOR of 2.49, 95% CI 1.32–4.71). Alike the bivariate analysis, with reference to the those who completed education up to college or above, the adjusted analysis also indicated that participants completing education up to high school or below were more likely to be LTFU (COR of 1.65, 95% CI 1.08–2.53, AOR of 2.01, 95% CI 1.10–3.66). Size of the social network was found to be significantly associated with LTFU in both crude (COR of 1.64, 95% CI 1.09–2.47) and adjusted (AOR of 1.75, 95% CI 1.09–2.80) analyses. Participants who were single/married, had bisexual sexual orientation and found their partner at pub/disco/tearoom/club showed positive associations with LTFU in the crude analyses but after adjusting for potential confounders, these associations were no longer significant in the final model (Table 2).

**Table 2:** Factors associated with loss-to-follow-up among MSM in Nanjing, China, 2008 (N = 410).

Characteristic		Crude		Adjusted	
		OR (95%CI)	*p-value*	OR (95%CI)	*p-value*
Age (years)		0.93(0.90–0.95)	<0.001	0.90(0.86–0.94)	<0.001
Cruising venue	*Internet*	*Ref*		*Ref*	
	*Pub- Disco- Tearoom- or Club*	2.57 (1.39- 4-74)	0.001	2.02(0.98–4.19)	0.061
	*Spa or bathhouse- Sauna*	0.86(0.49–1.51)	0.28	1.42(0.62–3.21)	0.92
	*Park- Public Restroom- or Public Lawn*	0.69(0.27–1.78)	0.21	1.12(0.36–3.47)	0.56
	*Other*	1.15(0.54–2.45)	0.92	2.05(0.86–4.88)	0.33
Marital status	*Divorced or Widowed*	*Ref*		*Ref*	
	*Single*	5.05(1.64–15.55)	0.002	2.11(0.50–8.81)	0.55
	*Married*	3.48 (1.06–11.40)	0.047	2.53(0.64–10.01)	0.20
Official resident (Hukou)	*Nanjing*	*Ref*		*Ref*	
	*Other cities in Jiangsu*	2.08(1.25–3.46)	0.005	1.50(0.85–2.65)	0.86
	*Other provinces*	3.97(2.26–6.98)	<0.001	2.49(1.32–4.71)	0.027
Education	*College or above*	*Ref*		*Ref*	
	*High school or below*	1.65(1.08–2.53)	0.021	2.01(1.10–3.66)	0.022
Income	*4000 or above*	*Ref*			
	*Less than 1000*	1.37(0.68–2.76)	0.32		
	*1000–4000*	1.12(0.59–2.13)	0.85		
Student	*Yes*	*Ref*			
	*No*	1.04(0.68–1.59)	0.86		
Sexual orientation	*Homosexual*	*Ref*		*Ref*	
	*Bisexual*	1.78(1.20–2.66)	0.004	1.34(0.85–2.12)	0.21
Unprotected anal sex	*No*	*Ref*			
	*Yes*	0.90(0.61–1.33)	0.60		
Unprotected vaginal sex	*No*	*Ref*			
	*Yes*	1.14(0.68–1.92)	0.63		
Reporting any STI related symptoms	*Yes*	*Ref*			
	*No*	1.08(0.69–1.69)	0.74		
Coverage of HIV/STI prevention services	*No*	*Ref*			
	*Yes*	0.74(0.41–1.33)	0.31		
HIV related knowledge	*No*	*Ref*			
	*Yes*	0.83(0.42–1.64)	0.60		
Size of social network	*10 or larger*	*Ref*		*Ref*	
	*Less than 10*	1.64(1.09–2.47)	0.018	1.75(1.09–2.80)	0.021
Syphilis	*Yes*	*Ref*			
	*No*	1.19(0.65–2.20)	0.57		

OR = Odds ratio 95% CI = 95% Confidence interval.

## Discussion

To the best of our knowledge this is the first study analyzing the role of socio-demographic and behavioral correlates of LTFU to BIs among MSM in China. In this cohort of 410 HIV sero-negative MSM attending a STI clinic for BIs and HIV testing in Nanjing, China during 2008, 221(53.9%) of the participants were LTFU during the 18-month period. This burden of LTFU was much higher than that observed in a 12-month follow-up study among MSM in Beijing (13.8%) during 2007 [33], in a cohort of MSM in Argentina (8.5%) during 2003–2004 [34] and in EXPLORE study where 14.2% of MSM enrolled from six US cities during 1999–2001 did not attend the 48-month follow-up visits [16]. It was much lower than that reported in the Smart Sex Quest study in US during 2002–2003, where approximately 85% of MSM did not have the complete follow-up data [35]. Even though MSM form a high risk population for HIV infection, it seems that perceived homophobia, ignorance about HIV, low self-perceived HIV risk and insensitivity of health care providers continues to limit access and uptake of these essential preventive services among this vulnerable population [1], [23], [36], [37].

In our study the chances of LTFU fell with age and participants who were official residents of other provinces in China had higher odds of drop out. These findings might be partially explained by China's massive and most extensive internal migration of younger population. Young, rural-to-urban male migrants have been recognized as the ‘tipping point’ for AIDS epidemic in China [38]. A subgroup of these migrants is known as ‘money boy’ who engage in same sex behavior mostly in MSM communities for earning money. They are mostly hidden, hard to reach population and vulnerable to HIV due to their high risk sexual behaviors [39]–[41]. A cross-sectional study among money boys in Shanghai, China demonstrated that compared to general male migrants, money boys had lower rate of condom use, more likely to have multiple partners, less knowledgeable about HIV/AIDS and only half of them were aware about free HIV counseling and testing [40]. Thus, a peer-driven counseling and behavioral intervention programs targeting these highly mobile and active MSM and their clients might be helpful in promoting HIV testing and safer sex among this at-risk population.

Consistent with the previous studies [33], [35], the participants in our study who completed the 18-month follow-up visit had higher level of education than who were LTFU. This study also revealed a positive association between lower level of education and LTFU which corroborated with a prior study among MSM in Beijing [33]. This might be due to the fact that MSM with less education are relatively less aware of the available HIV prevention services and probability of their participation being more affected by social stigma and discrimination might be another contributing factor.

The current study also indicated that small social network size was positively associated with LTFU. Prior research demonstrated a strong and consistent association between network characteristics and MSM sexual behavior [42], [43]. Constant vigilance by peer group, numerous social interconnections at individual and community levels, and peer driven counseling about the importance of HIV prevention interventions in a large social network were likely to influence the adherence to behavioral changes, counseling and HIV testing among MSM compared to relatively smaller social network [42], [44].

The present study had several limitations. Due to the potential lack of generalizability, extrapolation of results beyond the study sample was not recommended and any such effort should be made with caution. Although care was taken to avoid over-representation of participants linked with large social networks, chances of such bias might not be completely ruled out. Behavioral information was all self-reported and degree to which social desirability and accuracy of memory influenced these responses, remained questionable. We could not include too many variables in the final multivariate regression model, thus chances of residual confounding might still be there.

To conclude, MSM of younger age, having lower level of education, residing unofficially in Nanjing and being linked with smaller social network were all associated with higher likelihood of being LTFU. Despite of study limitations and scarcity of information on correlates of LTFU among MSM, we believe that the findings of our study stressed the need to better understand the specific characteristics of MSM who were vulnerable to LTFU so that a culturally competent, peer-driven, sustainable, multilevel collaborative approach might be designed for preventing or minimizing LTFU from such preventive efforts and HIV testing among these at risk MSM.
